# An Anabolic Signaling Response of Rat Soleus Muscle to Eccentric Contractions Following Hindlimb Unloading: A Potential Role of Stretch-Activated Ion Channels

**DOI:** 10.3390/ijms20051165

**Published:** 2019-03-07

**Authors:** Sergey Tyganov, Timur Mirzoev, Boris Shenkman

**Affiliations:** Myology Laboratory, Institute of Biomedical Problems RAS, 123007, 76A Khoroshevskoe shosse, 123007 Moscow, Russia; sentackle@yandex.ru (S.T.); bshenkman@mail.ru (B.S.)

**Keywords:** soleus muscle, hindlimb unloading, eccentric contractions, protein synthesis, anabolic signaling, stretch-activated channels

## Abstract

Mechanisms that convert a mechanical signal into a biochemical response in an atrophied skeletal muscle remain poorly understood. The aims of the study were to evaluate a temporal response of anabolic signaling and protein synthesis (PS) to eccentric contractions (EC) in rat soleus during hindlimb unloading (HU); and to assess a possible role of stretch-activated ion channels (SAC) in the propagation of a mechanical signal to mTORC1 following HU. Following HU, an isolated soleus was subjected to EC. Upon completion of EC, muscles were collected for western blot analyses to determine the content/phosphorylation of the key anabolic markers. We found that a degree of EC-induced p70S6K phosphorylation and the rate of PS in the soleus of 3- and 7-day unloaded rats was significantly less than that in control. A decrease in EC-induced phosphorylation of p70S6K, RPS6 and PS in the 7-day unloaded soleus treated with SAC inhibitor did not differ from that of the 7-day unloaded soleus without SAC blockade. The results of the study suggest that (i) HU results in a blunted anabolic response to a bout of EC, (ii) attenuation of mTORC1-signaling and PS in response to EC in unloaded soleus may be associated with inactivation of SAC.

## 1. Introduction

In both space physiology and rehabilitation medicine, it is critical to develop new effective exercise and pharmacological countermeasures in order to attenuate/prevent disuse-induced skeletal muscle atrophy as well as to enhance skeletal muscle recovery (regrowth) following a long period of inactivity. It is widely recognized that mechanical tension plays a key role in the regulation of skeletal muscle mass. Chronic mechanical loading results in an increase in muscle mass, while chronic mechanical unloading leads to muscle loss [1]. In order to understand how mechanical stimuli regulate muscle mass, it is important to comprehend how skeletal muscles sense mechanical signals and convert them into biochemical events (mechanotransduction) regulating the rate of protein synthesis (PS). It is now well-established that mammalian/mechanistic target of rapamycin complex 1 (mTORC1) plays a key role in the regulation of skeletal muscle PS and muscle mass in response to mechanical stimuli [2,3,4,5]. Phosphorylation of 70kDa ribosomal protein S6 kinase (p70S6K) on the Thr389 residue is typically used as a read-out of mTORC1-signaling. In addition to the mTORC1/p70S6K signaling, there are mTORC1-independent signaling pathways that are able to activate PS, in particular, glycogen synthase kinase-3β (GSK-3β)/initiation factor 2B (eIF2B) and extracellular signal-regulated kinase (ERK)/90kDa ribosomal protein S6 kinase (p90RSK) pathways [6,7]. It is important to note the role of stretch-activated ion channels (SAC) as possible mechanosensors of skeletal muscle fibers. These channels were first described in cultured skeletal muscle cells as mechanosensitive ion channels, which increase their open probability in response to mechanical stress [8]. It has been suggested that SAC are critical for a number of cellular processes, including electrolyte homeostasis and signal transduction [9,10]. SAC described in skeletal muscle appear to be permeable to Ca^2+^ and Na^+^ ions, and this action can be inhibited by gadolinium ions (Gd^3+^) [10]. However, to date, molecular mechanisms involved in sensing and converting an external mechanical signal into an anabolic response in an atonied/atrophied mammalian skeletal muscle remain undefined. We hypothesized that an eccentric contractions (EC)-induced anabolic response in rat soleus muscle following hindlimb unloading would be attenuated. Furthermore, this blunted anabolic response could be associated with impaired function of SAC. Therefore, the aims of the present study were (i) to evaluate a temporal response of anabolic signaling and protein synthesis (PS) to a bout of EC in the rat soleus at different time points of mechanical unloading and (ii) to assess a possible role of SAC in the propagation of a mechanical signal to mTORC1 in the soleus muscle following hindlimb unloading (HU).

## 2. Results

### 2.1. Experiment 1

#### 2.1.1. Body Weight, Soleus Weight to Body Weight Ratio and Mean Maximal Eccentric Tetanic Force (P_0_) Normalized to Muscle Cross-Sectional Area

There was no significant difference between the groups in body weight (Table 1). Soleus weight to body weight ratio significantly decreased by 16% (*p* < 0.05) after 7-day HS compared with the control animals. There was no significant difference between any groups in normalized P_0_, absolute tetanic tension was decreased after 7-day HS (Table 1).

#### 2.1.2. The Rate of Muscle Protein Synthesis

**Figure 1:**
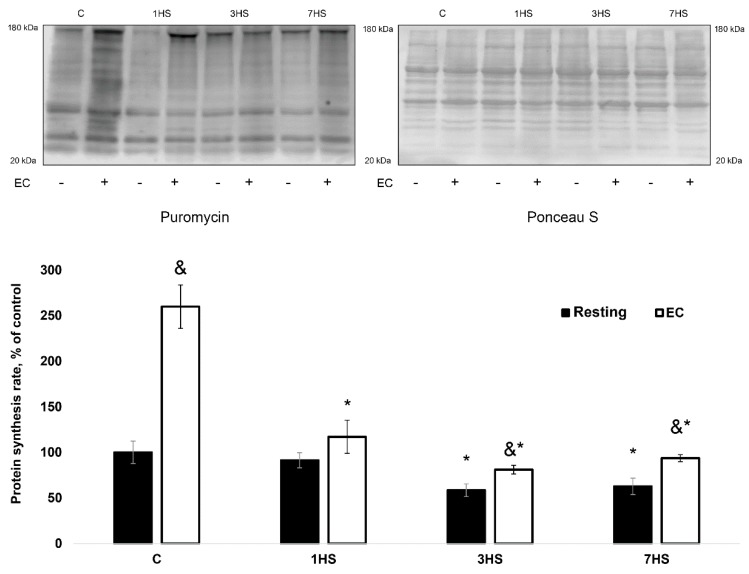
The rate of protein synthesis in the eccentrically-contracted rat soleus muscles following hindlimb unloading (experiment 1). Values are means ± SEM, expressed relative (%) to the control resting muscle; *n* = 7/group. C—control rats, 1HS, 3HS and 7HS—hindlimb unloading for 1, 3 and 7 days. The data were analyzed using 2-way ANOVA with *post hoc* Tukey. The main effect of HS *p* = 2.00459E-11, the main effect of EC *p* = 2.74866E-08, interaction effect *p* = 1.08513E-06; *—significant difference vs. C group (*p* < 0.05), &—significant difference from the resting muscle of the same group (*p* < 0.05). Black bars—resting muscle, white bars—eccentrically-contracted muscle.

The rate of PS in the resting soleus muscles significantly decreased by approximately 40% (*p* < 0.05) following 3- and 7 days of HS (Figure 1). A bout of EC of the isolated rat soleus resulted in a significant rise in PS vs. the resting muscle in the C, 3HS and 7HS groups (Figure 1).

However, the level of EC-induced PS increment in the 1HS, 3HS and 7HS groups was 81%, 77% and 64% lower (*p* < 0.05), respectively, than that in the C group (Figure 3a). 

#### 2.1.3. Phosphorylation of Key Signaling Proteins Involved in the Regulation of Muscle Protein Synthesis

**Figure 2:**
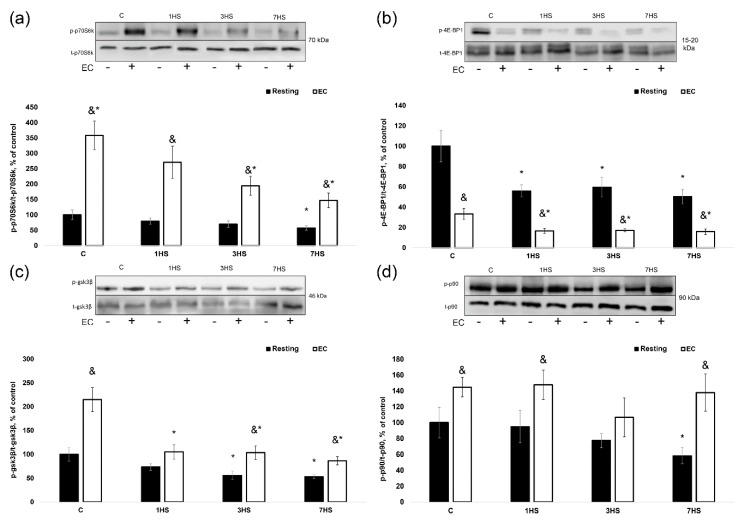
Phosphorylation status of the key anabolic signaling proteins in the eccentrically-contracted rat soleus muscles following hindlimb unloading (experiment 1). (**a**): p70S6K (Thr389) phosphorylation; (**b**): 4E-BP1 (Thr37/46) phosphorylation; (**c**): GSK-3β (Ser9) phosphorylation; (**d**): p90RSK (Thr359/Ser363) phosphorylation. Values are means ± SEM, expressed relative (%) to the control resting muscle; *n* = 7/group. C—control rats, 1HS, 3HS and 7HS—hindlimb unloading for 1, 3 and 7 days. The data were analyzed using 2-way ANOVA with *post hoc* Tukey. p70S6K (Thr389) phosphorylation: the main effect of HS *p* = 9,18811E-06, the main effect of EC *p* = 1,34515E-13, interaction effect *p* = 0,003124; 4E-BP1 (Thr37/46) phosphorylation: the main effect of HS *p* = 0,000353, the main effect of EC *p* = 2,00982E-10, interaction effect *p* = 0,021799; GSK-3β (Ser9) phosphorylation: the main effect of HS *p* = 1,01182E-08, the main effect of EC *p* = 5,21828E-08, interaction effect *p* = 0,005360; p90RSK (Thr359/Ser363) phosphorylation: the main effect of HS *p* = 0,125909, the main effect of EC *p* = 2,04532E-05, interaction effect *p* = 0,448363; *—significant difference vs. C group (*p* < 0.05), &—significant difference from the resting muscle of the same group (*p* < 0.05). Black bars—resting muscle, white bars—eccentrically-contracted muscle.

**Figure 3:**
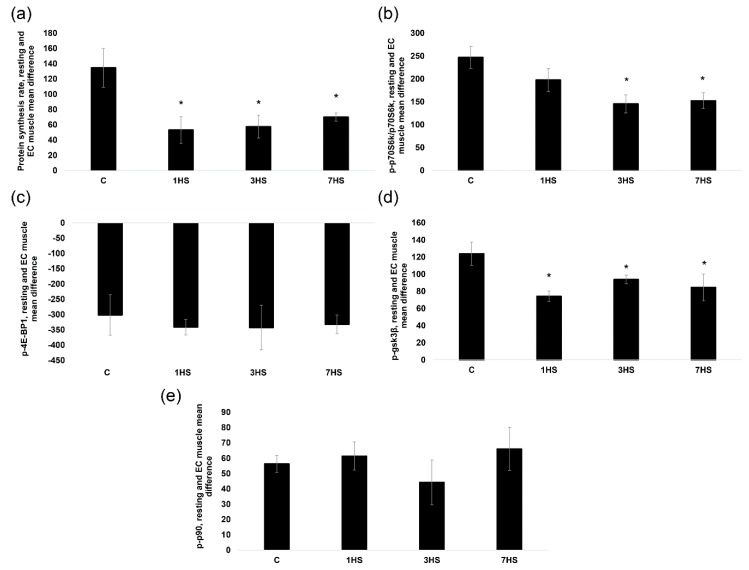
The mean difference in the rate of protein synthesis and phosphorylation of anabolic markers between resting and eccentrically-contracted muscles (experiment 1). (**a**): The rate of protein synthesis; (**b**): p70S6K (Thr389) phosphorylation; (**c**): 4E-BP1 (Thr37/46) phosphorylation; (**d**): GSK-3β (Ser9) phosphorylation; (**e**): p90RSK (Thr359/Ser363) phosphorylation. Values are means ± SEM; *n* = 7/group. C—control rats, 1HS, 3HS and 7HS—hindlimb unloading for 1, 3 and 7 days. *—significant difference vs. C group (*p* < 0.05). The data were analyzed using one-way ANOVA with *post hoc* Tukey. Protein synthesis: the main effect of HS *p* = 3,87195E-06; p70S6K (Thr389) phosphorylation: the main effect of HS *p* = 0,007496; 4E-BP1 (Thr37/46) phosphorylation: the main effect of HS *p* = 0,114387; GSK-3β (Ser9) phosphorylation: the main effect of HS *p* = 3,62852E-05; p90RSK (Thr359/Ser363) phosphorylation: the main effect of HS *p* = 0,381030; *—significant difference vs. C group (*p* < 0.05).

In the resting muscle, there was a significant 43% (*p* < 0.05) decrease in p70S6K phosphorylation in the 7HS group compared to the resting control (Figure 2a). p70S6K (Thr389) was significantly phosphorylated in response to EC in all groups, however EC-induced p70S6K phosphorylation increment in the 3HS and 7HS groups was significantly decreased by 104 and 97% (*p* < 0.05), respectively, vs. the C group (Figure 3b). 4E-BP1 (Thr37/46) phosphorylation was significantly decreased in all HS groups compared to the control group in resting muscles (Figure 2b). However, 4E-BP1 (Thr37/46) phosphorylation in response to EC was significantly lower in all groups, and the level of this decline did not differ between the groups (Figure 3c). In the resting isolated muscles of 3HS and 7HS, GSK-3β (Ser9) phosphorylation was 44 and 47% (*p* < 0.05) less than that in the resting control muscle (Figure 2c). A bout of EC revealed a significant decrease in GSK-3β phosphorylation increment in the isolated soleus muscles taken from hindlimb-unloaded rats as compared to the control rats (Figure 3d). 7-day HS resulted in a significant 42% (*p* < 0.05) decrease in p90RSK (T359 + S363) phosphorylation vs. the C group (Figure 2d) in resting soleus muscle. EC-induced increase in p90RSK phosphorylation was observed in the C group as well as in the 1HS and 7 HS groups, but this increased p90RSK phosphorylation was not statistically different among these groups (Figure 3e).

### 2.2. Experiment 2

#### 2.2.1. Body Weight, Soleus Weight to Body Weight Ratio and Mean Maximal Eccentric Tetanic Force (P_0_) Normalized to Muscle Cross-Sectional Area

Table 2 shows that there was no difference in body weight across the studied groups. The ratio of soleus weight to body weight significantly decreased after 7-day HS as compared to weight-bearing control rats. There was no significant difference between any groups in normalized P_0_ (Table 2), however absolute tetanic tension was decreased after 7-day HS (Table 2).

#### 2.2.2. The Rate of Muscle Protein Synthesis

**Figure 4:**
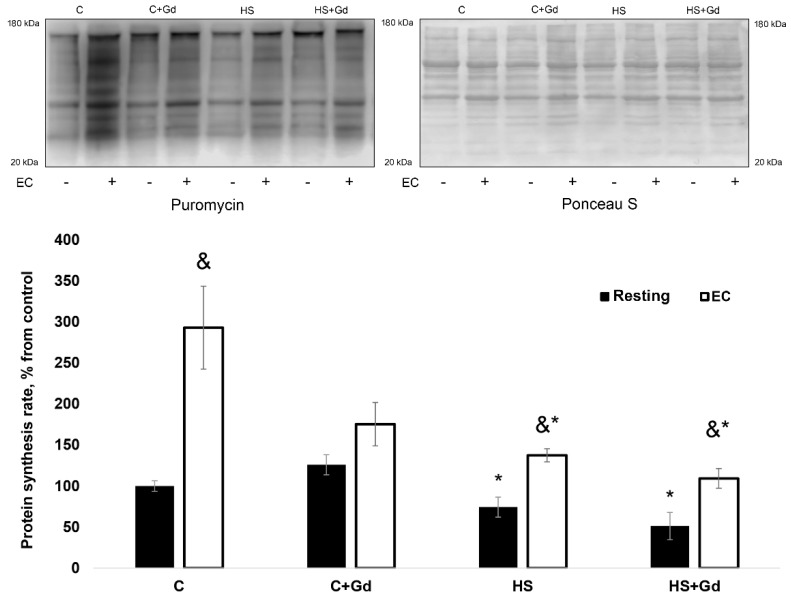
The effect of SAC blockade on the rate of protein synthesis in the eccentrically-contracted rat soleus muscles following 7-day hindlimb unloading (experiment 2). Values are means ± SEM, expressed relative (%) to the control resting muscle; *n* = 7/group. C—control animals, C + Gd—control animals with GdCl_3_ treatment of the isolated soleus, HS—animals subjected to 7-day hindlimb unloading, HS + Gd—hindlimb-unloaded animals with GdCl_3_ treatment of the isolated soleus. The data were analyzed using 3-way ANOVA with *post hoc* Tukey. The main effect of HS *p* = 1,51656E-06, the main effect of EC p=3,23095E-08, the main effect of Gd treatment *p* = 0,000187; interaction effect *p* = 0,000731; *—significant difference vs. C group (*p* < 0.05), &—significant difference from the resting muscle of the same group (*p* < 0.05). Black bars—resting muscle, white bars—eccentrically-contracted muscle.

As expected, the rate of PS in the resting muscles of the unloaded rats significantly declined (−40%, *p* < 0.05) compared to the control values (Figure 4). EC-induced increase in PS was 170% less (*p* < 0.05) in the C + Gd group vs. the C group. More importantly, EC-induced increase in PS was significantly less in the HS and HS + Gd groups vs. the C group (Figure 6a). There was no statistically significant difference between the HS and HS + Gd groups in terms of EC-induced increase in muscle PS (Figure 6a).

#### 2.2.3. Phosphorylation of Key Signaling Proteins Involved in the Regulation of Muscle Protein Synthesis

**Figure 5:**
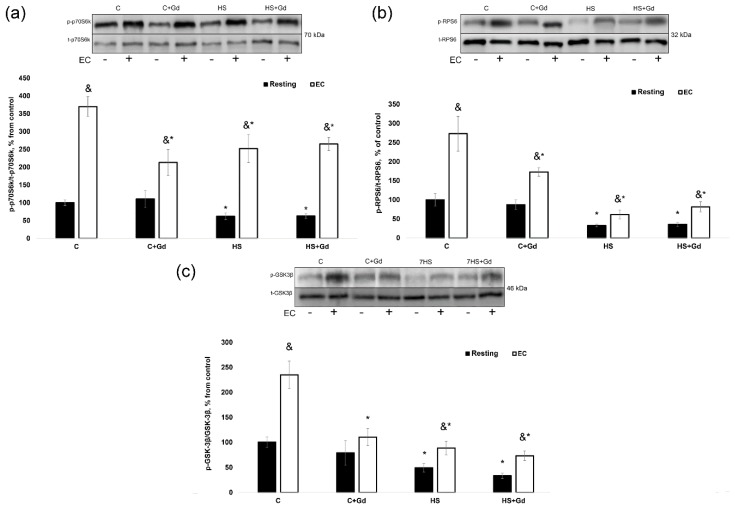
The effect of SAC blockade on the key markers of mTORC1-signaling and GSK-3β in the eccentrically-contracted rat soleus muscles following 7-day hindlimb unloading (experiment 2). (**a**): p70S6K (Thr 389) phosphorylation; (**b**): RPS6 (Ser 240/244) phosphorylation; (**c**): GSK-3β (Ser 9) phosphorylation. Values are means ± SEM, expressed relative (%) to the control resting muscle; *n* = 7/group. C—control animals, C + Gd—control animals with GdCl_3_ treatment of the isolated soleus, HS—animals subjected to 7-day hindlimb unloading, HS+Gd—hindlimb-unloaded animals with GdCl_3_ treatment of the isolated soleus. The data were analyzed using 3-way ANOVA with *post hoc* Tukey. p70S6K (Thr389) phosphorylation: the main effect of HS *p* = 0,001497, the main effect of EC *p* = 1,17298E-14, the main effect of Gd treatment *p* = 0,003678; interaction effect *p* = 0,001605; RPS6 (Ser 240/244) phosphorylation: the main effect of HS *p* = 2,40305E-11, the main effect of EC *p* = 4,53626E-09, the main effect of Gd treatment *p* = 0,000579; interaction effect p=0,000302; GSK-3β (Ser9) phosphorylation: the main effect of HS *p* = 1,26748E-06, the main effect of EC *p* = 4,4602E-07, the main effect of Gd treatment *p* = 0,003129; interaction effect *p* = 0,010773; *—significant difference vs. C group (*p* < 0.05), &—significant difference from the resting muscle of the same group (*p* < 0.05). Black bars—resting muscle, white bars—eccentrically-contracted muscle.

**Figure 6:**
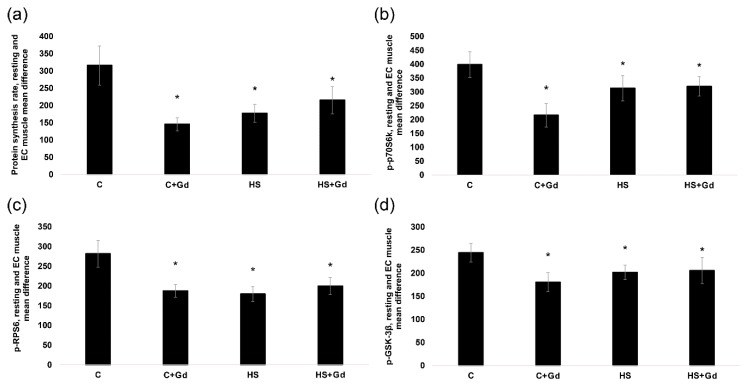
The mean difference in the rate of protein synthesis and phosphorylation of anabolic markers between resting and eccentrically-contracted muscles (experiment 2). (**a**): The rate of protein synthesis; (**b**): p70S6K (Thr389) phosphorylation; (**c**): RPS6 (Ser 240/244) phosphorylation; (**d**): GSK-3β (Ser9) phosphorylation. Values are means ± SEM; *n* = 7/group. C—control animals, C+Gd—control animals with GdCl_3_ treatment of the isolated soleus, HS—animals subjected to 7-day hindlimb unloading, HS+Gd—hindlimb-unloaded animals with GdCl_3_ treatment of the isolated soleus. The data were analyzed using 2-way ANOVA with *post hoc* Tukey. Protein synthesis: the main effect of HS *p* = 0,026604, the main effect of EC *p* = 0,008589, interaction effect *p* = 0,026372; p70S6K (Thr389) phosphorylation: the main effect of HS *p* = 0,001192, the main effect of EC *p* = 0,023999, interaction effect *p* = 0,011959; RPS6 (Ser 240/244) phosphorylation: the main effect of HS p=0,002087, the main effect of EC *p* = 0,020173, interaction effect *p* = 0,062066; GSK-3β (Ser9) phosphorylation: the main effect of HS *p* = 0,006279, the main effect of EC *p* = 0,001944, interaction effect *p* = 0,002417; *—significant difference vs. C group (*p* < 0.05).

In the isolated resting muscles, p70S6K (Thr389) phosphorylation was significantly decreased in the unloaded groups (HS and HS + Gd) vs. the C group (Figure 5a). In the EC muscles, the level of p70S6K (Thr389) phosphorylation increment was significantly down-regulated in C + Gd (−183%), HS (-85%) and HS+Gd (-79%) groups in comparison to the control values (Figure 6b). Similar changes in phosphorylation were observed for RPS6, a known p70S6K substrate. In the resting muscles, RPS6 phosphorylation was significantly declined in the HS and HS + Gd groups as compared to the C group (Figure 5b). The level of RPS6 (Ser 240/244) phosphorylation after a bout of EC was significantly diminished in C+Gd (−94%), HS (−102%) and HS + Gd (−82%) groups compared to the C group (Figure 6c). As for GSK-3β, a marker of mTORC1-independent anabolic pathway, we observed the same pattern of its Ser 9 phosphorylation as seen for p70S6K and RPS6 in resting and EC soleus muscles (Figure 5c and Figure 6d).

## 3. Discussion

To our knowledge, here we demonstrate for the first time a significant decrease in EC-induced PS rate as well as mTORC1-signaling in the isolated rat soleus muscle following 3 and 7 days of mechanical unloading. Moreover, our data suggest that attenuated anabolic response of the unloaded rat soleus could be associated, at least partially, with functional inactivation of SAC. The key finding of this study is that in the unloaded soleus muscle, both p70S6K (Thr389) phosphorylation and the rate of PS had a similar attenuated response to a bout of EC. This indicates that the first 1-3 days of unloading are enough to induce impaired transmission of a mechanical signal to PS in rat postural muscle. It is interesting to note, that, unlike other anabolic markers, 4E-BP1 phosphorylation in response to a bout of EC was reduced in all groups as compared to corresponding resting muscles. These data are consistent with Ato et al. (2016), who showed that immediately after EC the phosphorylation level of 4E-BP1 was significantly lower than that in the resting control muscle, while p70S6K (Thr389) phosphorylation was significantly higher compared to the resting control [11]. Apparently, 30 min period post EC was not enough to cause hyperphosphorylation of 4E-BP1. Mechanical unloading did not affect EC-induced p90RSK in the rat soleus muscle, indicating that ERK1/2 signaling pathway was not involved in the blunted anabolic response. As for GSK-3β, it can be suggested that the temporal pattern of its phosphorylation could be associated with a change in glycogen content that is known to increase in skeletal muscle fibers under unloading conditions [12]. However, the exact mechanism of GSK-3β activity regulation in response to EC during unloading remains unknown.

The obtained data concerning PS rate and phosphorylation of anabolic markers (p70S6K, p90RSK, GSK-3β) in the resting rat soleus generally agree with previously published reports of our laboratory [13,14] as well as other researchers [15,16,17,18].

A decrease in EC-induced anabolic response of the unloaded rat soleus muscle could be due to the impairment of both mechanosensory structures of the muscle fiber (such as SAC) and signaling molecules involved in mechanotransduction (such as focal adhesion kinase). In addition, an important role can be provided by the cytoskeleton in the transmission of a mechanical signal from the surface of the muscle fiber to anabolic regulatory proteins [19]. For example, HU-induced decrease in desmin content in rat soleus muscle [20,21], could interfere with a propagation of a mechanical signal to muscle PS. There is evidence that mechano-dependent activation of mTORC1 is directly mediated by phosphatidic acid, which is synthesized with the help of ζ-isoform of diacylglycerol kinase (DGK-ζ) [22].

Given that SAC are important skeletal muscle fiber mechanosensors [9], we further sought to assess a possible role of SAC in the propagation of a mechanical signal to mTORC1 and PS in the soleus muscle following 7-day hindlimb unloading. It has been previously been shown that SAC blockade with gadolinium or streptomycin can lead to a decrease in muscle fibers membrane depolarization [23,24] as well as a decrease in the degree of rat tibialis anterior hypertrophy following repeated eccentric contractions [23]. Furthermore, SAC appear to be necessary for full EC-induced activation of AKT and p70S6K in rat tibialis anterior muscle [25]. In the current study, a significant decrease in the phosphorylation level of the key mTORC1 targets and GSK-3β in the isolated rat soleus muscle in response to EC was observed both in the HS group and the HS+Gd group. Since the inhibition of SAC with Gd^3+^ did not lead to further decline in the EC-induced phosphorylation of the key anabolic markers (GSK-3β, p70S6K, RPS6) compared to the HS group, our data indicate that 7-day hindlimb unloading alone can induce SAC downregulation with subsequent attenuation of mTORC1-signaling and PS.

Previously it has been shown that a bout of EC results in a significant elevation of intracellular Ca^2+^ in rat skeletal muscle and that SAC may play a permissive role in this process [26]. Stretch-activated Ca^2+^ ion channels contribute to the Ca^2+^ influx into myofibers which activates calmodulin [27]. Calmodulin interacts with the human vacuolar protein sorting-34 (hVPS34) and activates mTORC1 in the presence of essential amino acids [28]. Zanou et al. (2012) identified a Ca^2+^-dependent activation of the PI3K/Akt/mTOR/p70S6K pathway during myoblast differentiation and skeletal muscle regeneration [29]. Although the exact mechanisms for how calcium ions may affect anabolic signaling pathways in skeletal muscle are still undefined, there is evidence that an overload-induced transient receptor potential cation channel subfamily V member 1 (TRPV1)—mediated increase in intracellular Ca^2+^ concentration can lead to the activation of mTORC1 [30]. Importantly, this load-induced elevation in Ca^2+^ concentration was not completely prevented in Trpv1-null myotubes, suggesting that other ion channels (such as SAC) might also be involved in load-induced increase in Ca^2+^ levels and subsequent mTORC1 activation. It is also worth noting that in embryonic kidney cells it has been shown that initial priming step for p70S6K activation is calcium-dependent, and failure of this priming step to occur results in a global reduction of p70S6K phosphorylation [31]. Therefore, it is possible that unloading-induced attenuation of SAC function could contribute to the blunted anabolic response of the isolated rat soleus to mechanical stimuli (eccentric contractions). However, elucidation of the precise molecular mechanism(s) underlying transmission of a mechanical signal from SAC to mTORC1 and muscle protein synthesis awaits further investigation.

## 4. Materials and Methods

### 4.1. Ethical Approval

All procedures with the animals were approved by the Biomedicine Ethics Committee of the Institute of Biomedical Problems of the Russian Academy of Sciences/Physiology section of the Russian Bioethics Committee (protocol no. 421, 14.04.2016 and protocol no. 444, 28.03.2017). All experiments were performed in strict accordance with the guidelines and recommendations in the Guide for the Care and Use of Laboratory Animals of the National Institutes of Health. All efforts were made to minimize animal suffering and discomfort. Animals were housed in a temperature-controlled room on a 12:12-h light-dark cycle with food pellets and water provided ad libitum.

### 4.2. Animals and Hindlimb Unloading

Male 2.5 month old Wistar rats weighing 225 ± 10 g were obtained from the certified Nursery for laboratory animals of the Institute of Bioorganic Chemistry of the Russian Academy of Sciences (town of Pushchino, Moscow region, Russian Federation). Mechanical unloading was simulated using a standard hindlimb unloading (HU) model [32], following the recommendations provided by the European Convention for the protection of Vertebrate Animals used for Experimental and Scientific purposes (Council of Europe number 123, Strasbourg, 1985). In all experiments, prior to all surgical procedures, animals were anaesthetized with an intraperitoneal injection of tribromoethanol (240 mg/kg^−1^). The depth of anesthesia was evaluated by testing the pedal withdrawal reflex (toe and foot pad pinch). After muscle excision, the rats were euthanized by a tribromoethanol overdose (I.P.) followed by cervical dislocation.

#### 4.2.1. Experiment 1

Twenty-eight male Wistar rats weighing 225 ± 10 g were randomly assigned to the following 4 groups (*n* = 7/group): vivarium cage control (C), hindlimb unloading for 1 day (1HS), 3 days (3HS) and 7 days (7HS). Following HS, an isolated soleus muscle from the right hindlimb was placed in an organ culture medium and subjected to a bout of EC. An isolated soleus muscle from the left hindlimb was also placed in an organ culture medium but was not subjected to EC (resting muscle).

#### 4.2.2. Experiment 2

Twenty-eight male Wistar rats weighing 225 ± 10 g were randomly assigned to the following 4 groups (*n* = 7/group): 1) vivarium cage control (C), 2) control rats an isolated soleus of which was incubated with 20 µM GdCl_3_ (Santa Cruz Biotechnology, Santa Cruz, CA, USA, cat # sc-224004), SAC inhibitor (C + Gd^3+^), 3) hindlimb unloading for 7 days (HS), 4) hindlimb unloaded rats an isolated soleus, of which was incubated with 20 µM GdCl_3_, SAC inhibitor (HS + Gd^3+^). Upon HS completion, isolated soleus muscles from the right hindlimb were subjected to EC, while isolated soleus muscles from the left hindlimb remained resting in chilled oxygenated Krebs–Henseleit buffer.

### 4.3. Eccentric Contractions of the Isolated Rat Soleus Muscle

**Figure 7:**
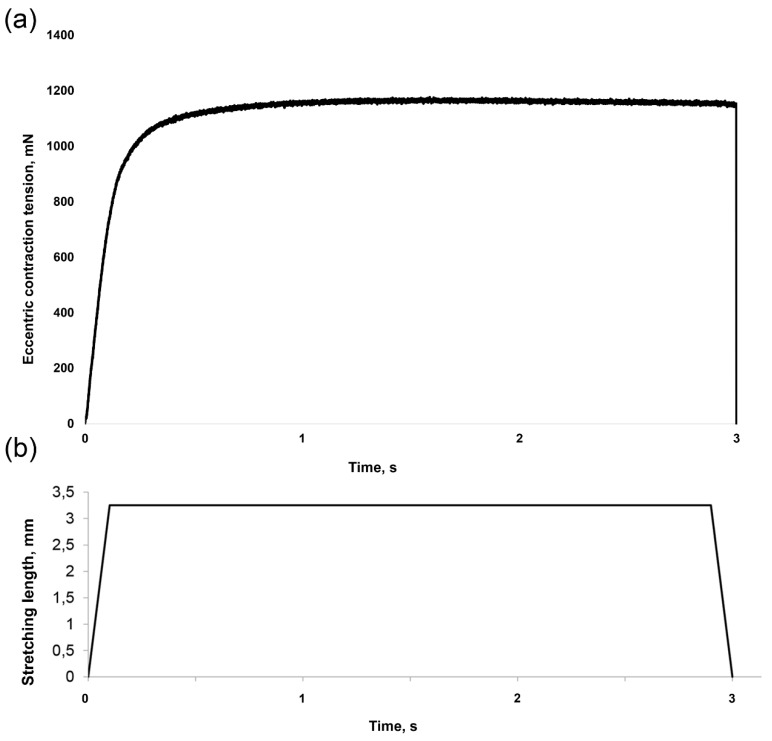
Representative force and muscle length tracing from one typical eccentric contraction. (**a**): changing in muscle force. (**b**): changing in muscle length.

Rat soleus muscles were isolated from the hindlimbs and mounted between a lever arm of a position feedback servomotor (Aurora Scientific, Ontario, Canada) and an immovable pin in an organ bath and maintained at 37 °C with a thermo-regulated water-jacket. The bath contained Krebs–Henseleit buffer (120mM NaCl, 4.8mM KCl, 25mM NaHCO_3_, 2.5mM CaCl_2_, 1.2mM KH_2_PO_4_, 2mM MgSO_4_, 5mM Hepes) supplemented with 25mM glucose and equilibrated with a 95% O_2_, 5% CO_2_ gas. For the experiment 2, 20 µM [26,33] of GdCl_3_ was added to the medium. EC protocol was adopted from O’Neil et al. (2009) [34]. Muscles were plunged into Krebs–Henseleit buffer for 15 min before all contractions. Eccentric contractions were evoked by electrically field stimulating the soleus muscle with a 80 V 50 Hz pulse for 3 s with a 1 ms square wave. L_0_ was measured during muscle dissection and evaluated with single twitch contractions followed by length adjustment. Left muscle was resting at L_0_ while right soleus was subjected to EC-contractions. The stimulation produced about 80% of the muscle’s isometric maximum contraction, determined from preliminary experiments. At the onset of this electrical stimulus, the soleus muscle was lengthened 15% using a 100 ms ramp and then held at this length for the remainder of the contraction. At the end of the contraction, the soleus muscle was returned to optimal length using a 100 ms ramp. Each contraction was followed by a 10 s rest period during which time the muscle was maintained at L_0_. After the sixth repetition of contractions, there was an additional 50 s of rest period. This pattern of stimulation was repeated for a total of 10 sets of six repetitions, resulting in 60 contractions over a 22 min period. L_0_ was constantly checked during all sets of contractions in order to avoid possible knot slippage. Upon completion of the bout of eccentric contractions, the muscles were maintained at L_0_ for an additional 30 min [34] and then collected for biochemical analysis. Signals from the force transducer/dynamometer and electrical stimulator were controlled and monitored with DMC/DMA software (Aurora Scientific, Toronto, Ontario, Canada). Maximal eccentric contraction force (P_0_) was measured and normalized to muscle cross-sectional area (CSA). CSA was evaluated as muscle wet weight divided by the product of muscle optimal length and density (1.07 g cm^−3^) [35,36]. Representative force and muscle length tracing from one typical eccentric contraction is shown in (Figure 7).

### 4.4. SUnSET Technique for Measuring the Rate of PS

SUnSET (surface sensing of translation) is a nonradioactive technique that allows to measure protein synthesis in skeletal muscle. This technique involves the use of the antibiotic puromycin (a structural analogue of tyrosyl-tRNA), and anti-puromycin antibodies to detect the amount of puromycin incorporation into nascent peptide chains. It was shown that when puromycin is used at low concentrations (40 nmol/g), the accumulation of puromycin-conjugated peptides accurately reflects the rate of protein synthesis [37]. The SUnSET technique uses standard Western blotting and immunohistochemical technologies to visualize and quantify the rates of protein synthesis [38]. For measurements of protein synthesis, rats were given an intraperitoneal injection of 0.04 μmol/g puromycin hydrochloride (Enzo Life Sciences, Farmingdale, NY, USA) dissolved in PBS. Rat soleus muscles were extracted at exactly 15 min after I.P. puromycin injection.

### 4.5. Western Blot Analysis

The skeletal muscle tissue (30 mg) was homogenized in the ice-cold lysis buffer: 50 mM Tris (pH 7.4), 150 mM NaCl, 1% Nonidet P-40, 0.5% sodium deoxycholate, 0.1% SDS, 0.004% sodium azide, and 5 mM EDTA, supplemented with 1 mM DTT, 1 mM PMSF, 10 μg/mL leupeptin, 5 μL/mL pepstatin and 1% aprotinin (Sigma-Aldrich, St. Louis, MO, USA), mammalian protease inhibitor cocktail (Amresco, Solon, OH, USA), and phosphatase inhibitor cocktail B (Santa Cruz Biotechnology, Santa Cruz, CA, USA). The muscle lysates were incubated for 20 min at 4 °C and then centrifugated for 10 min at 12000 g. Protein concentration was quantified using Bradford protein assay (Bradford, 1976). Bovine serum albumin was used as a standard. The samples were diluted in Laemmli buffer. The total protein (20–50 μg) was subjected to SDS-PAGE [39], and the proteins were then transferred to nitrocellulose membrane (Bio-Rad Laboratories, Hercules, CA, USA). Then, to verify equal loading of protein in all lanes, the nitrocellulose membrane was dyed by Ponceau S. The membranes were blocked for 1 h at room temperature with the blocking buffer (4% nonfat milk powder; TBS, pH 7.4; and 0.1% Tween 20) and incubated overnight at 4°C with primary antibodies (diluted in TBS-T) against p-p70S6K (Thr 389) (1:2000; Santa Cruz Biotechnology, Santa Cruz, USA, sc-11759) and p70s6k (1:1000, Cell Signaling Technology, Beverly, MA, USA, #9202), p-4E-BP1 (Thr37/46) (1:1000, Cell Signaling Technology, Beverly, MA, USA, #2855,) and 4E-BP-1 (1:1000, Cell Signaling Technology, Beverly, MA, USA, #9452), p-p90RSK1 (T359+S363) (1:1000, Abcam, Cambridge, MA, USA, ab32413) and p90RSK-1 (1:1000, Santa-Cruz Biotechnology, Beverly, MA, USA, sc-231), p-GSK-3β (Ser 9) (1:1000, Cell Signaling Technology, Beverly, MA, USA, #9322) and GSK-3β (1:1000, Cell Signaling Technology, Beverly, MA, USA, #12456), P-S6RP (S240/244) (1:1000, Cell Signaling Technology, Beverly, MA, USA, #5364), S6RP (1:1000, Cell Signaling Technology, Beverly, MA, USA, #2217), puromycin (1:3000, Kerafast Inc., Boston, USA, EQ0001), GAPDH (1:10000, Applied Biological Materials Inc., Richmond, British Columbia, Canada, no. G041). Three 10-min washes with TBS-T were then performed. After that, the membranes were incubated for 1 h at room temperature with horseradish peroxidase-conjugated secondary antibodies to rabbit immunoglobulins (1:30000, Santa Cruz Biotechnology, Santa Cruz, CA, USA, sc-2004). For detection of puromycin-labeled proteins secondary goat anti-mouse IgG (H + L)-HRP conjugate antibodies (1:35000; Bio-Rad Laboratories, Hercules, CA, USA, #1706516) were used. The membranes were then washed again in TBS-T 3 times for 10 min and incubated in Immun-Star HRP Chemiluminescent system (Bio-Rad Laboratories, Hercules, CA, USA, #1706516). The protein bands were quantified using C-DiGit Blot Scanner (LI-COR Biotechnology, Lincoln, NE, USA) and Image Studio Digits software. Following image capture of phosphorylated proteins, membranes were stripped of the phosphospecific antibodies, using RestoreTM Western Blot Stripping Buffer (Thermo Scientific, Waltham, MA, USA), for 30 min at 37 °C after which the membranes were re-probed with primary antibodies for each respective total protein. The signal from the phospho-protein was normalized to the total protein. For protein synthesis detection, the measurements of the chemiluminescent signals were performed by determining the density of each whole lane with the entire molecular weight range of puromycin-labeled peptides. Phospho and total blots were done on the same gel. Each gel contained samples from all groups. Protein samples were run at least in duplicate on the same gel. The representative blots are of the same samples (phospho and total). Total protein staining (Ponceau S) and GAPDH protein expression were used as loading controls.

### 4.6. Statistical Analysis

All data are expressed as means ± SEM. Two-sided t-tests were applied to determine differences between the resting and contracting muscle. Statistical analysis of the remaining data was performed using one-way ANOVA, two-way ANOVA (eccentric contractions · hindlimb unloading) or three-factorial ANOVA (eccentric contractions · hindlimb unloading · gadolinium injection) as indicated in the appropriate figure legends. Tukey’s post hoc analysis was used to determine differences when interactions existed. Differences with values of *p* < 0.05 were considered to be statistically significant.

## 5. Conclusions

The results of the study suggest that (i) hindlimb unloading results in a blunted anabolic response of the isolated rat soleus to a bout of EC, and (ii) attenuation of mTORC1-signaling and PS in response to EC in the unloaded soleus muscle may be associated with functional inactivation of SAC.

## Figures and Tables

**Table 1:** Changes in rat’s body weight, soleus weight-to-body weight ratio and P_0_, and P_0_ normalized to muscle cross-sectional area following 1-, 3- and 7 days of hindlimb unloading.

Group	Rat Weight, g	Soleus Weight, mg/Rat Weight, g	P_0_, mN	P_0_/Cross-Sectional Area, N/cm^2^
C	220 ± 8	0.40 ± 0.011	802.4 ± 24.3	19.4 ± 1.4
1HS	214 ± 3	0.42 ± 0.013	744.5 ± 26.1	18.2 ± 1.1
3HS	222 ± 10	0.372 ± 0.015	742.5 ± 21.7	20.2 ± 1.0
7HS	210 ± 7	0.336 ± 0.018 *	660.8 ± 24.9 *	20.2 ± 1.1

Values are means ± SEM. Values are means ± SEM, expressed relative (%) to the control resting muscle; *n* = 7/group. C—control rats, 1HS, 3HS and 7HS—hindlimb unloading for 1, 3 and 7 days. The data were analyzed using one-way ANOVA with *post hoc* Tukey. Rat weight: the main effect of HS *p* = 0.158784, soleus weight/rat weight ratio: the main effect of HS *p* = 0.001586, P_0_: the main effect of HS *p* = 0.002901, P_o_/CSA: the main effect of HS *p* = 0.589732; *—significant difference vs. C group, *p* < 0.05.

**Table 2:** Changes in rat’s body weight, soleus weight-to-body weight ratio and P_0_, and P_0_ normalized to muscle cross-sectional area following 7 days of hindlimb unloading (experiment 2).

Group	Rat Weight, g	Soleus Weight, mg/Rat Weight, g	P_0_, mN	P_0_/Cross-Sectional Area, N/cm^2^
C	225 ± 16	0.43 ± 0.03	940.2 ± 33.3	23.5 ± 1.7
C + Gd	220 ± 10	0.44 ± 0.04	911.4 ± 15.7	19.4 ± 1.2
HS	226 ± 8	0.31 ± 0.03 *	715.1 ± 23.9 *	23.6 ± 1.1
HS + Gd	225 ± 7	0.35 ± 0.03 *	740.6 ± 15.2 *	21.3 ± 2.0

Values are means ± SEM, *n* = 7/group. C—control animals, C + Gd—control animals with GdCl_3_ treatment of the isolated soleus, HS—animals subjected to 7-day hindlimb unloading, HS + Gd—hindlimb-unloaded animals with GdCl_3_ treatment of the isolated soleus. The data were analyzed using 2-way ANOVA with *post hoc* Tukey. Rat weight: the main effect of HS *p* = 0,278046, the main effect of Gd *p* = 0,834042; interaction effect *p* = 0,345367; soleus weight/rat weight ratio: the main effect of HS *p* = 3,31084E-10, the main effect of Gd *p* = 0,059001; interaction effect *p* = 0,020047; P_o_: the main effect of HS *p* = 1,76513E-4, the main effect of Gd *p* = 0,234589; interaction effect *p* = 0,009768; P_o_/CSA: the main effect of HS *p* = 0,546122, the main effect of Gd *p* = 0,216795; interaction effect *p* = 0,619122; *—significant difference vs. C group; *p* < 0.05.

## Abbreviations

4E-BP1	Eukaryotic translation initiation factor 4E-binding protein 1
CSA	Cross-sectional area
DGK-ζ	Diacylglycerol Kinase ζ
EC	Eccentric contractions
eIF2B	Eukaryotic initiation factor 2B
ERK1/2	Extracellular signal-regulated kinase 1/2
GSK-3β	Glycogen synthase kinase 3β
HU	Hindlimb unloading
L_0_	Optimal muscle length
mTORC1	Mammalian target of rapamycin complex 1
P_0_	Maximal muscle contraction tetanic force
p70S6K	Ribosomal protein S6 kinase p70
PS	Protein synthesis
RPS6	S6 ribosomal protein
SAC	Stretch-activated ion channels
SUnSET	Surface sensing of translation
